# Antioxidative behavior of a2-macroglobulin in intervertebral disc degeneration

**DOI:** 10.5937/jomb0-39557

**Published:** 2023-03-15

**Authors:** Yuhong Chen, Huaixiang Wei, Feng Xu

**Affiliations:** 1 Chun'an County Hospital of Traditional Chinese Medicine, Department of Orthopedics, Hangzhou, China; 2 Xiangxi Tujia and Miao Autonomous Prefecture People's Hospital, Department of Pain, Jishou, China; 3 Boji Hospital Changxing Zhejiang Province, Huzhou, China

**Keywords:** antioxidant, nucleus pulposus cells, intervertebral disc degeneration, a2-macroglobulin, extracellular matrix, antioksidans, nukleus pulposus ćelije, degeneracija intervertebralnog diska, a2-makroglobulin, ekstracelularni matriks

## Abstract

**Background:**

To clarify if a2-macroglobulin (α2M) has an antioxidative effect during the progression of the intervertebral disc degeneration (IVDD).

**Methods:**

The content of α2M and reactive oxygen species (ROS) were measured to compare mildly and severely degenerated human nucleus pulposus (NP) tissue by immunohistochemistry, mass spectrometry, and enzyme-linked immunosorbent assay (ELISA). Additionally, exogenic α2M was used to culture severely degenerated NP tissue in vitro. The effects of α2M on hypochlorite (HOCl)-treated NP cells were evaluated, containing antioxidative enzymes, ROS level, collagen II, and aggrecan expression, MMP3/13, and ADAMTS4/5.

**Results:**

ROS level increased in severely degenerated NP, accompanying with a decreased α2M content. Supplement of α2M could decrease the ROS level of cultured NP in vitro, meanwhile, the MMP13 and ADAMTS4 expression were also reduced. It was found that treatment of HOCl resulted in oxidative damage to NP cells and decreased α2M expression in a dose and time-dependent manner. Furthermore, exogenic α2M stimulation reversed the HOCl-triggered ROS accumulation. The promotion of SOD1/2, CAT, GPX1, collagen II, and aggrecan, and suppression of MMP3/13, ADAMTS4/5 expression caused by α2M were also observed.

**Conclusions:**

Our study indicates that α2M has an antioxidative ability in degenerated NP cells by promoting the antioxidative enzyme production.

## Abstract (sr)

**Uvod:**

Cilj rada je bio da razjasni da li a2-makroglobulin (a2M) ima antioksidativni efekat tokom progresije degeneracije intervertebralnog diska (IVDD).

**Metode:**

Izmeren je sadržaj a2M i reaktivnih vrsta kiseonika (ROS) da bi se uporedilo blago i teško degenerisano ljudsko nukleus pulposus (NP) tkivo imunohistohemijom, masenom spektrometrijom i enzimskim imunosorbentnim testom (ELISA). Pored toga, egzogeni a2M je korišćen za kultivaciju teško degenerisanog NP tkiva in vitro. Procenjeni su efekti a2M na NP ćelije tretirane hipohloritom (HOCl), koje sadrže antioksidativne enzime, nivo ROS, kolagen II i ekspresiju agrekana, MMP3/13 i ADAMTS4/5.

**Rezultati:**

Povećan nivo ROS kod teško degenerisanog NP, praćen je smanjenim sadržajem a2M. Dodatak a2M mogao bi smanjiti nivo ROS kultivisanog NP in vitro, u međuvremenu, ekspresija MMP13 i ADAMTS4 je takođe smanjena. Utvrđeno je da tretman HOCl dovodi do oksidativnog oštećenja NP ćelija i smanjuje ekspresiju a2M na način koji zavisi od doze i vremena. Štaviše, egzogena a2M stimulacija je preokrenula akumulaciju ROS-a izazvanu HOCl. Takođe je primećena promocija SOD1/2, CAT, GPKS1, kolagena II i agrekana i supresija ekspresije MMP3/13, ADAMTS4/5 izazvane a2M.

**Zaključak:**

Naša studija pokazuje da a2M ima antioksidativnu sposobnost u degenerisanim NP ćelijama promovišući proizvodnju antioksidativnih enzima.

## Introduction

The intervertebral disc has no blood supply and consists of a peripheral annulus fibrosis, a central nucleus pulposus (NP), and upper and lower endplates [1]. NP has fewer cells but abundant extracellular matrix (ECM), such as collagen II and aggrecan, which are normally secreted by NP cells [2]. Intervertebral disc degeneration (IVDD) is mainly manifested in the imbalance in the synthesis and degradation of cellular and ECM components. Some proteolytic enzymes can degrade the ECM of intervertebral discs, of particular interest, are matrix metalloproteinases (MMPs), a disintegrin and metalloproteinase with thrombospondin motifs (ADAMTS), which promote abnormal apoptosis of NP cells and cause further degeneration of the intervertebral disc [3] [4].

α2-macroglobulin (α2M) is an ancient, evolutionarily conserved polymer glycoprotein, which has a variety of active forms and complex functional effects involving in the regulation and transport of substances in the body [5]. Especially when people realized the broad-spectrum anti-protease effect of α2M, it was more fully applied in motor system diseases such as acromion bursitis, tendinitis [6], osteoarthritis [7], and intervertebral disc disease [8]. In addition to the ability of protease inhibitor in eliminating excessive endogenous and exogenous proteases, it has also been confirmed in anti-radiation and anti-tumor [9] effects. Recently, more and more reports mention α2M involving in the inhibition of reactive oxygen species (ROS) [10]. However, whether α2M related to the mediation of oxidative stress of IVDD is not fully understood. Oxidative stress refers to the serious imbalance between the generation of oxygen radicals and the antioxidant defense, leading to the accumulation of ROS in the body or cells, which leads to cytotoxicity and tissue damage [11]. ROS can cause degradation of ECM by inhibiting collagen II and aggrecan synthesis and destroying ECM structure. As people get older, ROS accumulates, and the level ofoxidative stress in the body gradually increases, which leads to the disability of intervertebral disc cells, inflammatory infiltration, and ECM disorders that resulting in IVDD [12].

Our present study aimed to clarify the role of α2M in IVDD, especially its antioxidative behavior,using cultured NP tissue and NP cells *in vitro*. Our finding provides a new understanding of α2M in the therapeutic strategy of IVDD that containing oxidative stress balance.

## Materials and methods

### Source of patient samples

To clarify the difference of α2M in NP tissues of different degenerated degree, 16 NP samples were collected from patients undergoing disc herniation operations, which were grouped into mildly degenerated group and severely degenerated group based on the Pfirrmann score [13] (Grade II or III belongs to the mild group; Grade IV or V belongs to the severe group). This research project was approved by the Ethics Committee of our hospital.

### NP tissue treatment

We conserved the tissues in cold sterile dulbecco’s modified eagle medium (DMEM) medium (Gibco, Rockville, MD, USA) immediately after cutting from the patients and transferred to the lab for tissue culture *in vitro*. We used different concentrations of α2M from human plasma (Sigma-Aldrich, St. Louis, MO, USA) to culture the severely degenerated NP tissue for 3 days and collected for the following experiment.

### NP cells isolation and treatment

The tissues were minced and digested with type II collagenase (0.2%) and trypsin (0.15%) solution at 37°C overnight. We filtrated the cell solution, centrifuged and resuspended in DMEM/F-12 medium (Gibco, Rockville, MD, USA) containing 10% fetal bovine serum (FBS) (Gibco, Rockville, MD, USA). We used different concentrations of hypochlorous acid (HOCl) resulting in direct oxidative damage to NP cells. Additionally, α2M from human plasma (Sigma-Aldrich, St. Louis, MO, USA) was used to reverse HOCl.

### Hematein eosin staining (HE)

NP tissue was first treated as follows: fixed with 4% formaldehyde, dehydrated with a gradient of alcohol, embedded in paraffin, and cut into 5 μm thick slices. Sections were then dewaxed, hydrated, hematoxylin stained for 5 min, 0.7% hydrochloric acid ethanol differentiated for 5 s, and eosin-stained for 30 s.

### Immunohistochemical (IHC)

Sections were suffered as follows: dewaxed, hydrated, and blocked with 10% goat serum for 1 hour. Sections were then incubated with α2M (ab58703, Abcam, Cambridge, MA, USA) primaryantibody overnight at 4°C. After incubated with biotinylated IgG and Elite ABC reagent (Beyotime, Shanghai, China), sections finally were developed by 3, 3-diaminobenzidine and counterstained with hematoxylin.

### Enzyme-linked immunosorbent assay (ELISA)

The levels of α2M, MMP13, and ADAMTS4 in by NP tissue or NP cells were analyzed by ELISA kit (ab108888; ab100605; ab213753, Abcam, Cambridge, MA, USA) according to the manufacturer’s instructions.

### ROS measurement

ROS content of NP tissue or NP cells was determined using *in vitro* ROS/RNS assay (Cell Biolabs, San Diego, CA, USA) according to the manufacturer’s instructions. The relative fluorescence units (RFU) at an excitation/emission wavelength of 488/525 nm was measured by a microplate reader.

### Reverse transcription-polymerase chain reaction (RT-PCR)

Total RNA was extracted from NP cells by TRIzol reagent (Invitrogen, Carlsbad, CA, USA) and reverse-transcribed to cDNA templates by PrimeScript™ RT Mix (RR036A, TaKaRa, Tokyo, Japan). RT-PCR assay was performed to assay relative gene expression containing superoxide dismutase1 (SOD1), SOD2, catalase (CAT), glutathione peroxidase1 (GPX1), MMP3, MMP13, ADAMTS4, and ADAMTS5 by normalization of glyceraldehyde 3-phosphate dehydrogenase (GAPDH) according to 2^−ΔΔCt^ methods. The primers of the genes were listed in Table 1.

**Table 1:** Primer sequences of the genes for RT-PCR. RT-PCR, quantitative reverse-transcription polymerase chain reaction

Gene name	Forward (5’>3’)	Reverse (5’>3’)
SOD1	GGTGGGCCAAAGGATGAAGAG	CCACAAGCCAAACGACTTCC
SOD2	GGAAGCCATCAAACGTGACTT	GCGTTGATGTGAGGTTCCAG
CAT	TGGAGCTGGTAACCCAGTAGG	CCTTTGCCTTGGAGTATTTGGTA
GPX1	CAGTCGGTGTATGCCTTCTCG	GAGGGACGCCACATTCTCG
MMP-3	AGTCTTCCAATCCTACTGTTGCT	TCCCCGTCACCTCCAATCC
MMP13	ACTGAGAGGCTCCGAGAAATG	GAACCCCGCATCTTGGCTT
ADAMTS4	GAGGAGGAGATCGTGTTTCCA	CCAGCTCTAGTAGCAGCGTC
ADAMTS5	GAACATCGACCAACTCTACTCCG	CAATGCCCACCGAACCATCT
GAPDH	ACAACTTTGGTATCGTGGAAGG	GCCATCACGCCACAGTTTC

### Immunofluorescence (IF) staining

After treatment, NP cells were washed with phosphate buffered saline (PBS), fixed with 4% formaldehyde, and blocked with 5% bovine serum albumin (BSA). NP cells were following incubated with collagen II and aggrecan primary antibodies (Abcam, Cambridge, MA, USA) overnight at 4°C. Followed incubated with goat anti-rabbit IgG antibody (Beyotime, Shanghai, China) for 1 h at room temperature. Nucleus was stained with 4’,6-diamidino-2-phenylindole (DAPI), and the positive fluorescence was visualized by the fluorescence microscope.

### Statistical analysis

Statistical analysis was performed using Statistical Product and Service Solutions (SPSS) 22. 0 software (IBM, Armonk, NY, USA). Data were displayed as the means ± standard deviations (SD) with triplicated experiments. Differences between two groups were analyzed by using the Student’s t-test. Comparison between multiple groups was done using One-way ANOVA test followed by Post Hoc Test (Least Significant Difference). *P*<0.05 was considered statistically significant.

## Results

### α2M level decreased in degenerated NP tissue

**Figure 1:**
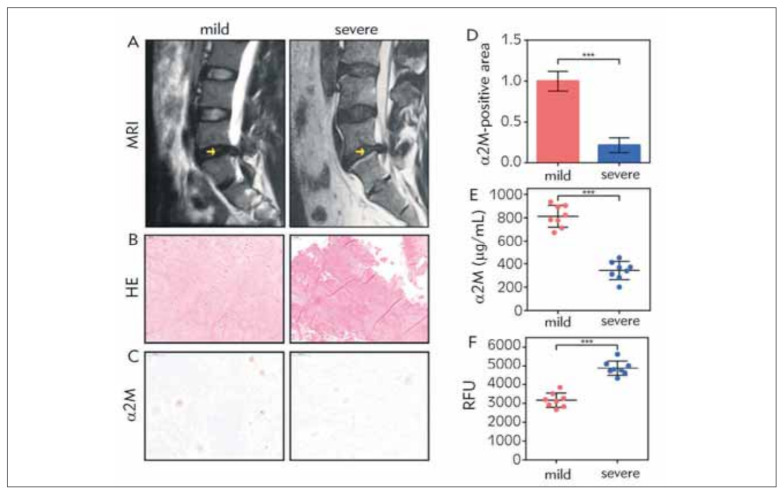
α2M level decreased in degenerated NP tissue. Representative images of (A) MRI, the yellow arrows indicated the operation section, (B) HE staining (magnification: 200×) (C) IHC targeting α2M of both mildly and severely degenerated NP tissue. (magnification: 200×) (D) Quantification analysis of IHC. NP tissue from the 16 patients was lysed to measure (E) α2M with ELISA methods and (F) total ROS level. The values are mean ± SD of three independent experiments. (***P<0.001)

To clarify the relation of α2M level with the NP tissue’s degenerated degree, we collected 8 mildly and severely degenerated NP tissue based on the Pfirrmann score, separately. As shown in Figure 1A, the yellow arrows indicated the operation section, which was the source of our NP specimen. The height of the severely degenerated NP tissue was lower than the mild one, and the border between the NP and the annulus fibrosus was more blurred than the mild one. From the HE staining, we can see that the arrangement of ECM in the severe degeneration group is more chaotic, and the NP cells are more hypertrophic compared to the mild degeneration group (Figure 1B). We used two methods to analyzed the α2M level in NP tissue of the two groups containing IHC staining (Figure 1C and Figure 1D) and ELISA (Figure 1E), the results of which indicated that α2M expression significantly reduced in severely degenerated condition compared to the mild group. Additionally, we also measured the total ROS expression and found the severe group had a higher ROS level, as expected (Figure 1F). Therefore, degenerated NP tissue normally expresses a lower α2M level but a higher ROS level as the degree of degeneration increases.

### α2M supplement suppressed ROS of degenerated NP tissue in vitro

**Figure 2:**
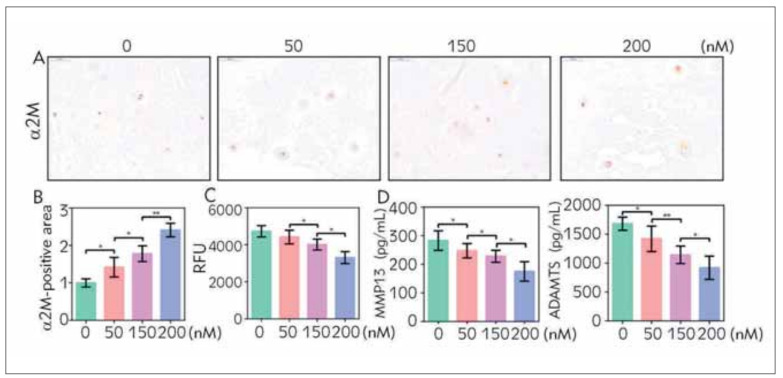
α2M stimulation decreased ROS, MMP13, and ADAMTS4 of severely degenerated NP tissue. We cultured severely degenerated NP tissue in α2M (50, 150, 200 nmol/L) growth medium for 3 days. The protein expression level of α2M was determined by (A) IHC (magnification: 200×) and (B) quantification analysis. (C) Total ROS level of NP tissue. (D) The content of MMP13 and ADAMTS4 was assayed by ELISA. The values are mean ± SD of three independent experiments. (*P<0.05, **P<0.01)

To determine the antioxidative effect of α2M on the NP cells, we used α2M from human plasma to culture the severe degeneration NP tissue. After 3 days, the α2M expression in NP cells of the severe group was upregulated in a dose-dependent manner resulting from the exogenic α2M stimulation (Figure 2A and Figure 2B). Surprisingly, α2M was efficient to suppress the accumulation of ROS along as the increased α2M expression (Figure 2C). As a widely used anti-protease, we also tested the content of MMP13 and ADAMTS4 after the application of α2M. The result of ELISA indicated that α2M significantly decreased the MMP13 and ADAMTS4 expression in dose-dependent (Figure 2D). The data suggest that α2M acts not only an anti-protease but also an antioxidant in the degenerated NP tissue.

### α2M supplement suppressed HOCl-induced ROS of degenerated NP cells in vitro

**Figure 3:**
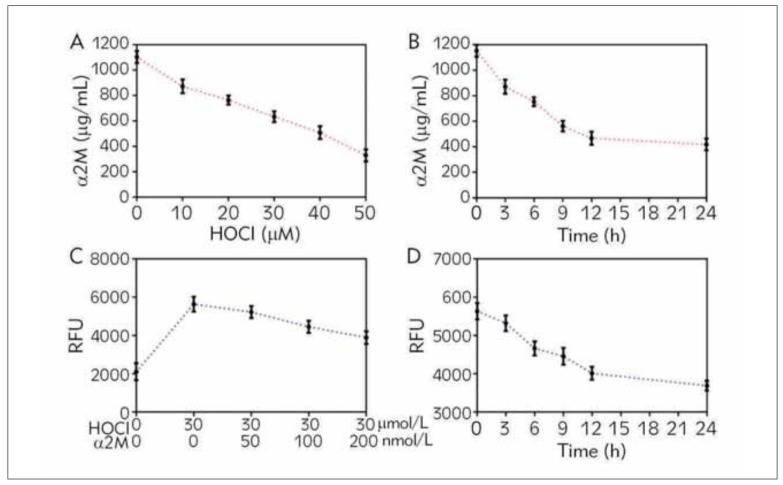
α2M stimulation reversed HOCl-induced oxidative stress of NP cells. NP cells of mildly degenerated NP tissue were treated with HOCl (from 10 to 50 μmol/L) for 6 h, or treated with 30 μmol/L from 3 h to 24 h; Besides, NP cells were pretreated with 30 μmol/L HOCH for 6 h and then cultured with α2M (from 50 to 200 nmol/L) for another 24 h, or cultured with 200 nmol/L α2M from 3 h to 24 h. (A, B) The protein expression level of α2M was determined by ELISA. (C, D) Total ROS level of NP cells. The values are mean ± SD of three independent experiments.

To further elucidate the effect of abundant oxidative stress on the expression of α2M and the role of α2M in the oxidative stress state, we isolated NP cells from the mild degeneration tissue and used HOCl to activate the reactive oxygen and caused NP cells oxidative stress. As shown in Figure 3A, HOCl gradually decreased the α2M expression with the increased concentration from 10 μmol/L to 50mmol/L. In addition to this, HOCl affected the α2M level in a time-dependent manner as well, which presented a sharp drop in the first 12 hours (Figure 3B). Furthermore, we cultured the HOCL-pretreated NP cells with exogenic α2M from 50 nmol/L to 200 nmol/L, and the total ROS gradually decreased caused by the stimulation of α2M (Figure 3C). Besides, the suppression of ROS based on a time-dependent manner was also observed (Figure 3D). These results indicate that the accumulation of ROS caused by HOCl truly affects the α2M expression of NP cells, however, the supplement of α2M also rejects the HOCl-induced oxidative stress of NP cells.

### α2M supplement protected antioxidative enzymes expression in HOCl-treated NP cells

**Figure 4:**
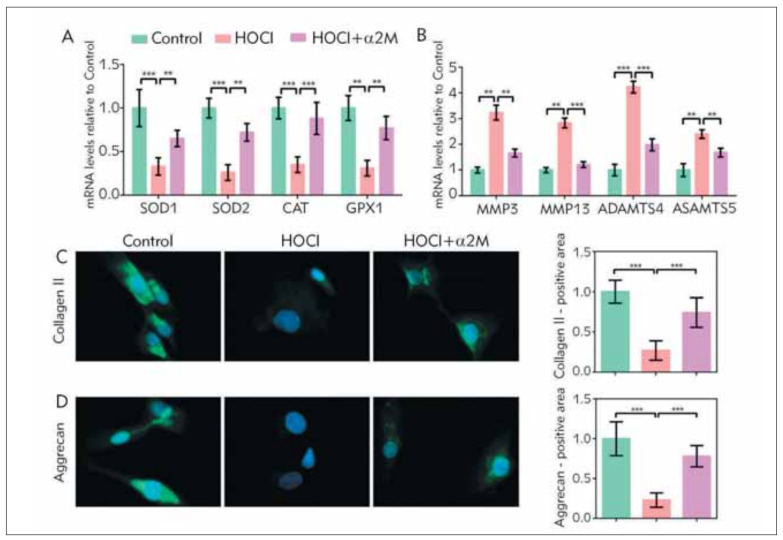
α2M level decreased in degenerated NP tissue. Representative images of (A) MRI, the yellow arrows indicated the operation section, (B) HE staining (magnification: 200×) (C) IHC targeting α2M of both mildly and severely degenerated NP tissue. (magnification: 200×) (D) Quantification analysis of IHC. NP tissue from the 16 patients was lysed to measure (E) α2M with ELISA methods and (F) total ROS level. The values are mean ± SD of three independent experiments. (***P<0.001)

The accumulation of ROS results from the imbalance between the generation of oxygen radicals and the antioxidant. Therefore, we concerned whether α2M played a role in the regulation of antioxidative enzymes such as SOD, CAT, and GPX. We pretreated NP cells with HOCl to trigger the vast ROS, which obviously reduced the mRNA expression of SOD1, SOD2, CAT, and GPX1 (Figure 4A). However, the exogenic stimulation of α2M effectively promoted these antioxidative enzymes expression, suggesting the antioxidative effect of α2M might be related to the promotion of antioxidative enzymes. The disorder of ECM is the main character of IVDD containing the loss of collagen II and aggrecan. MMPs and ADAMTS regulate the dynamic balance of ECM and can degrade different ECM components. We found the excessive ROS could increase the MMP3, MMP13, ADAMTS4, and ADAMTS5 mRNA level, which also was reversed by the α2M treatments (Figure 4B). Finally, the protein expression of collagen II (Figure 4C) and aggrecan (Figure 4D) was determined by IF. HOCl significantly decreased the collagen II and aggrecan compared to the control. After using the α2M, the collagen II and aggrecan content were upregulated again compared to the HOCl group. Therefore, we think α2M plays a role in the promotion of antioxidative enzymes and inhibition of protease, which leads to the protection of collagen II and aggrecan synthesis of NP cells.

## Discussion

α2M was originally used as a protease inhibitor to clear exogenous and excessive endogenous proteases from tissues, which contains almost all types of proteases [14]. In addition to being a broad-spectrum protease inhibitor, α2M has many other functions: as a carrier of some small molecules such as cytokines and growth factors, including TGF-β, TNF, IFNγ, PDGF [15], FGF, IL-6 [16], and NGF; regulate cell apoptosis [17]; regulate thrombin activity and fibrin hydrolysis [18]; regulate cell proliferation, adhesion, and migration [19]; regulation of oxidative stress, which is one of the pathology of IVDD. The toxic effects of oxidative stress on the body are manifested in lipid peroxidation of biological membranes, denaturation of intracellular proteins and enzymes, and DNA damage, causing abnormal cell metabolism and eventually leading to cell death or apoptosis [20]. ROS is a class of free radicals closely related to oxidative stress, mainly including superoxide anions (O_2_^-^), hydroxyl radicals (·OH), H_2_O_2_, NO, and HOCl, which can be produced by the reaction of mitochondria and catalase in cells and can also be induced under special physical and chemical environments [21].

To uncover the relation of α2M in the IVDD, we tested the α2M expression in NP tissue of different degradation from 16 patients. The findings indicated α2M decreased along with the IVDD. To further clarify the antioxidative function of α2M in the IVDD, we cultured the NP tissue with α2M, which presented a ROS inhibition compared to the control. Apart from this, we also isolated NP cells and established an oxidative stress model by HOCl. HOCl is a strong oxidant in ROS, that mainly produced by activated neutrophils and macrophages. It has a strong bactericidal effect and its bactericidal ability is about 50 times that of H_2_O_2_ [22]. The basic level of HOCl is positive for the body to resist the invasion of foreign pathogens, but when the level of HOCl is too high, it will induce oxidative stress to cause damage to the body [23]. Hence, we used HOCl to cause the oxidative stress in NP cells as well as the reduction of α2M, which was consistent with the previous study [11]. Hopefully, the additional supplement of α2M performed an excellent effect on the inhibition of ROS caused by HOCl with a dose and time-dependent.

Apart from the excessive ROS, the weak activity of antioxidative enzymes also contributes to the destruction of the oxidative balance in cells. Therefore, we also analyzed the gene expression of SOD1/2, CAT, GPX1 under the treatment of HOCl and α2M. Despite the continuous production of ROS, the body can still fight oxidative stress through an antioxidant system, mainly including enzymatic and non-enzymatic mechanisms to antagonize the oxidation response. The main endogenous enzymatic antioxidants are SOD, CAT, GPX, peroxidase, glutathione reductase, among which SOD and CAT are the main anti-ROS enzymes [24]. They together form the body’s antioxidant defense system. The balance between ROS and the antioxidant defense system is a necessary condition for maintaining the homeostasis of the disc [25]. In our study, HOCl suppressed the SOD1/2, CAT, and GPX1 mRNA expression, but α2M showed a positive effect on their expression,which strengthened the antioxidative force in thedegenerated NP cells. The antiprotease activity ofα2M has been fully confirmed [26]. Wang et al. [7] found α2M inhibited MMP13 expression in the prevention of posttraumatic osteoarthritis. Tortorella et al. [27] reported α2M was an endogenous inhibitor of ADAMTS4 and ADAMTS5 in osteoarthritis. In this present study, α2M was verified to suppress the MMP and ADAMTS both in the severely degenerated NP tissue and in the HOCl-treated NP cells. Therefore, the antioxidative and antiprotease effect of α2M contributes to the protection of collagen II and aggrecan expression, which is meaningful to the prevention of IVDD.

## Conclusions

In conclusion, α2M participates in the development of IVDD, and it not only has anti-inflammatory effects but also has the antioxidative behavior relating to the upregulation of the antioxidative enzyme production. However, its specific mechanism still needs further research. α2M as a therapeutic drug has good clinical application prospects, but obtaining α2M with high purity, large yield, reasonable price, and more safety is also the direction that needs to be worked in the future.

## Dodatak

### Conflict of interest statement

All the authors declare that they have no conflict of interest in this work.
